# Physicochemical, Bioactive, Sensory, and Microbial Evaluation of Functional Drinking Yoghurt Fortified With Beetroot and Orange Juice

**DOI:** 10.1002/fsn3.72452

**Published:** 2026-10-04

**Authors:** Md Masudul Islam Khan, Md Noitik Ali, Ayshee Tabassum, Devpriya Bhattacharjee, Shahin Alam, Md Shahedul Islam

**Affiliations:** ^1^ Department of Nutrition and Food Engineering Daffodil International University Dhaka Bangladesh

**Keywords:** drinking yoghurt, fruit juice, functional food, immune booster, nutritional enhancement, plant‐fortified dairy beverage

## Abstract

This study aimed to formulate and quality‐characterize drinking yoghurt with beetroot pulp, abundant in antioxidants, rich in betalains and phenolic compounds and to enhance its nutritional, bioactive, and sensory attributes by adding orange juice. In this study, three different mixtures of beetroot pulp and orange juice were used: Sample A: 240 g yoghurt, 30 g beetroot pulp, and 30 mL orange juice; Sample B: 225 g yoghurt, 45 g beetroot pulp, and 30 mL orange juice; and Sample C: 255 g yoghurt, 15 g beetroot pulp, and 30 mL orange juice. Formulation‐dependent differences were observed in the measured bioactive parameters. Sample B showed the highest DPPH radical‐scavenging activity at 800 ppm (56.1% ± 2.1%) and the lowest estimated IC_50_ (429.4 ppm), together with the highest TPC (51.4 ± 1.5 mg GAE/100 g) and TFC (14 ± 1 mg CE/100 g). The sensory analysis showed that sample A achieved the highest mean sensory scores across the evaluated attributes, whereas Sample C received the lowest taste score. During 0–35 days of refrigerated storage, no coliforms or *Escherichia coli* were detected in any sample, that is, < 1 CFU/mL yoghurt, whereas the pH of all drinking yoghurt samples remained relatively stable, ranging from 4.0 to 4.5, with sample B demonstrating the highest pH and sample C the lowest. The findings demonstrate formulation‐dependent differences in sensory and bioactive characteristics and support further investigation of beetroot‐orange fortified drinking yoghurt using independently replicated production batches and broader shelf‐life assessment.

## Graphical abstract

Bioactive, sensory, and physicochemical properties of beetroot pulp and orange juice‐fortified drinking yoghurt demonstrated formulation‐dependent differences among the three formulations. Sample B exhibited the maximum seedling DPPH radical‐scavenging activity and the maximum phenolic and flavonoid content, while Sample A had the maximum sensory scores. Refrigerated storage for 35 days resulted in pH levels in the range of 4.0–4.5, and no coliforms or *Escherichia coli* were detected among the formulations, thus signifying the opportunity of using beetroot‐orange fortified drinking yoghurt as a functional dairy‐based product.

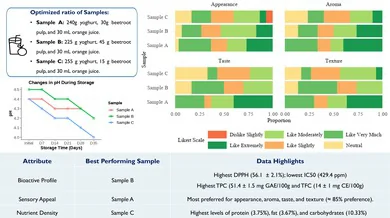

## 1 Introduction

In today's fast‐paced world, people seek better health with little hassle. Yoghurt is a widely consumed fermented dairy product with established nutritional value and broad consumer acceptance. Yoghurt has been part of the human diet for several millennia and goes by many names worldwide. The word “yoghurt” is believed to have come from the Turkish word “yoğurmak,” which means to thicken, coagulate, or curdle (Fisberg and Machado 2015). Yoghurt is one of the best‐known foods that contains probiotics. Yoghurt is defined by the *Codex Alimentarius* of 1992 as a coagulated milk product that results from the fermentation of lactic acid in milk by *Lactobacillus bulgaricus* and *Streptococcus thermophilus* (Adolfsson et al. 2004). Yoghurt provides proteins, lipids, minerals, and other nutrients and can also serve as a suitable food matrix for incorporating plant‐derived bioactive compounds. Its live beneficial bacteria support gut health, immunity, and overall well‐being. Today, people want more from food—functional options with natural antioxidants to fight oxidative stress. Demand for such products has surged; many now turn to fruits and vegetables for protection from aging and disease. Adding beetroot and orange juice to yoghurt boosts its functionality. *Beta vulgaris* subsp. *Vulgaris* L. (beetroot), a Chenopodiaceae root, ranks among the top 10 antioxidant vegetables due to its betalains, phenolic acids, saponins, alkaloids, steroids/triterpenes, catechins, and flavonoids (Pandita et al. 2020). These compounds have been associated with antioxidant activity and may contribute to the protection of cellular components from oxidative damage. Beetroot is therefore increasingly investigated as a functional ingredient in fortified food products.

Beta‐carotene is a fat‐soluble pigment in red, orange, and yellow vegetables and fruits. These compounds work synergistically to neutralize free radicals, reduce inflammation, and even improve blood flow by boosting nitric oxide levels, which has earned beetroot a spot in athletes' diets for enhancing performance. The bioactive compounds present in beetroot may contribute to antioxidant activity by reducing oxidative stress and protecting cellular components such as lipids, proteins, and DNA from oxidative damage (Mirmiran et al. 2020). Beetroot is also rich in its high nitrate content, which the body converts to nitric oxide for better cardiovascular health, and its folate for cell repair, making it a valuable source of bioactive ingredients for fortified foods (Mirmiran et al. 2020). The body converts beta‐carotene to vitamin A when needed. Beta‐carotene acts as an antioxidant, blocking damaging reactive oxygen species. Eating beta‐carotene‐rich foods has been linked to cancer prevention (Johnson and Russell 2010). Its antioxidants defend cells from damage by ROS or free radicals, which can disrupt metabolism, damage DNA, and impair lipids and proteins (Pandita et al. 2020). Numerous animal and laboratory studies have substantiated Beta‐carotene's ability to inhibit tumor cell growth and the progression of carcinogenesis (Patrick 2000).

On the other hand, orange is a major crop and an important source of bioactive compounds with health‐promoting properties. Over the years, more than 100 carotenoids, many of them tentatively identified, have been reported in the peel and pulp of different citrus fruits (Antonio et al. 2007). These bioactive compounds, alongside generous vitamin C, flavonoids, and limonoids, deliver potent anti‐inflammatory and immune‐boosting effects; for instance, studies link regular orange consumption to lower stroke risk and improved skin health by protecting collagen from UV damage (Mirmiran et al. 2020). Eating beta‐carotene‐rich foods has been linked to cancer prevention through mechanisms such as enhanced detoxification enzymes and inhibited tumor cell proliferation, as shown in numerous animal and laboratory studies (Lung 2003). The incorporation of beetroot and orange juice into yoghurt may enhance its nutritional value and functional properties.

However, while plenty of studies have examined the addition of whole fruits or vegetables to fermented dairy products, plant‐enriched dairy products have been widely investigated; limited studies have evaluated combined beetroot pulp and orange juice incorporation into drinking yoghurt, according to the existing literature. Beetroot supplies dietary nitrates (Sanlier et al. 2026), betalains (Milton‐Laskibar et al. 2021), and phenolics (Stoica et al. 2025), while orange juice supplies vitamin C, flavonoids, and carotenoids (Aschoff et al. 2015; Miles and Calder 2021). The combination of these ingredients in a fermented dairy product could affect physicochemical stability, sensory acceptability, bioactive retention, and microbial quality. Therefore, this study aimed to characterize three drinking yoghurt formulations containing a constant amount of orange juice and different proportions of beetroot pulp, with emphasis on proximate composition, selected bioactive indices, sensory acceptance, pH changes during refrigerated storage, and selected microbiological indicators.

## 2 Materials and Methods

### 2.1 Raw Materials and Reagents

Fresh roots of beetroot ( *Beta vulgaris* L.) and fruits of orange ( *Citrus sinensis* ) at commercial maturity were procured on the day of processing from a certified local market in Dhaka, Bangladesh, and brought to the laboratory as quickly as possible under refrigerated conditions (4°C–8°C) to prevent deterioration in quality. Cow's milk (full‐fat, 3.5%–4.0% fat) was obtained from a single local dairy supplier to reduce variability between batches. All samples were visually inspected; damaged, overripe, and diseased units were discarded following standard quality selection procedures described for fortified yogurts (Molina et al. 2019).

Analytical‐grade chemicals and reagents for physicochemical and antioxidant analyses, including β‐carotene standard, DPPH (2,2‐diphenyl‐1‐picrylhydrazyl), acetone, petroleum ether, ethanol, and sodium sulfate, were supplied by Sigma‐Aldrich (USA) or equivalent, in accordance with best practice in dairy and carotenoid research. All the microbiological media (Plate Count Agar, Violet Red Bile Agar, MacConkey Agar, EMB Agar, MRS Agar, and EC Broth) were supplied by HiMedia Laboratories India and prepared as per the manufacturer's instructions/ISO (Molina et al. 2019).

### 2.2 Preparation of Beetroot Pulp and Orange Juice

Beetroot roots were washed under running tap water to remove soil and debris, then rinsed with distilled water and peeled with stainless steel knives to avoid metallic contamination. Peeled beetroot was cut into 2–3 cm cubes and blanched in boiling water (approximately 100°C) for 2 min to inactivate peroxidase and polyphenol oxidase, following procedures commonly used to stabilize color and carotenoids. Blanched cubes were cooled rapidly in ice water, drained, and homogenized in a laboratory blender with distilled water in a 4:1 (w/w) ratio until a uniform pulp was obtained; the pulp was passed through a stainless‐steel sieve to remove coarse fiber (Zhao et al. 2024).

Sweet orange fruits were washed, surface‐sanitized with 100 ppm chlorinated water, rinsed with potable water, and peeled by hand. Juice was obtained from a mechanical juicer, filtered with double‐layer muslin cloth to remove seeds and coarse pulp, before being stored at 4°C in amber bottles under light‐absent conditions to minimize the light‐induced vitamin C and carotenoid degradation until use (Molina et al. 2019).

### 2.3 Preparation of Starter Culture and Milk

A commercial lyophilized yoghurt starter containing *Streptococcus thermophilus* and *Lactobacillus delbrueckii* ssp. bulgaricus was pre‐cultured in sterile reconstituted skim milk at 42°C for 16–18 h until pH 4.5, following the manufacturer's instructions and protocols used in probiotic and carotenoid‐enriched yogurts. The active culture was stored at 4°C for up to 24 h before use (Molina et al. 2019).

Raw cow's milk was filtered through muslin cloth, standardized to the desired fat content if necessary, and heated to 85°C–95°C for 5 min (high‐heat treatment) to denature whey proteins and improve water‐holding capacity, consistent with methods used in high‐quality stirred and fortified yogurts. Heated milk was rapidly cooled to inoculation temperature (42°C–44°C) in an ice‐water bath while being gently stirred (Molina et al. 2019).

### 2.4 Formulation of Enriched Drinking Yoghurt

The activated yoghurt starter (at 2%, v/v) was inoculated into the pasteurized milk base and incubated in a temperature‐controlled incubator at 40°C–42°C until pH reached 4.5 ± 0.1. The pH was monitored at 30‐min intervals using a calibrated pH meter. Fermentation times were based on the end points recommended for probiotic and functional yogurts to maintain appropriate acidity, as well as microbial survival (Molina et al. 2019).

The set yoghurt was gently stirred to obtain a uniform stirred yoghurt base. Beetroot pulp and orange juice were then incorporated into the yoghurt to prepare three formulations:

- Sample A: 240 g yoghurt + 30 g beetroot pulp + 30 mL orange juice
- Sample B: 225 g yoghurt + 45 g beetroot pulp + 30 mL orange juice
- Sample C: 255 g yoghurt + 15 g beetroot pulp + 30 mL orange juice

Preliminary studies were conducted to identify concentrations that would offer acceptable sensory attributes and ensure product stability and homogeneity, and the formulation levels were established accordingly. The selected concentrations were low (15 g), medium (30 g), and high (45 g) levels of beetroot pulp integration. The concentration of orange juice was kept constant to minimize variability related to acidity and flavor intensity.

The formulations were homogenized with a laboratory stirrer at 300–500 rpm for 3–5 min to achieve a homogeneous distribution of plant ingredients without inducing excessive air entrapment. The enriched drinking yogurts were dispensed into sterilized, hermetically sealed glass bottles (250 mL), chilled to 4°C, and held under refrigeration until analysis (Molina et al. 2019).

### 2.5 Proximate Composition

The proximate composition was measured using AOAC Official Methods to ensure internationally comparable and acceptable results.

- Moisture content: Determined by oven drying at 105°C until constant weight, according to AOAC 925.09; approximately 5 g of sample was spread on pre‐weighed aluminum dishes and dried for three to four hours, cooled in a desiccator, and reweighed (Molina et al. 2019).
- Ash content: Samples were ashed in a muffle furnace at between 550°C and 600°C for 4 to 6 h (AOAC 923.03) until light gray ash was produced; crucibles were cooled in a desiccator before being weighed (Molina et al. 2019).
- Protein content: Determined by the Kjeldahl method (AOAC 920.87), samples were subjected to digestion with concentrated sulfuric acid and catalyst, distillation, and titration, the total nitrogen content being multiplied by 6.38 (dairy protein factor) (Molina et al. 2019).
- Fat content: Determined using the Gerber method (AOAC 2000) employing sulfuric acid and amyl alcohol in butyrometers, followed by centrifugation and reading of the fat column at 65°C (Patel et al. 2019).
- Carbohydrate content: Determined by difference as 100 – (moisture + ash + protein + fat) following fortified yoghurt studies (Patel et al. 2019).

### 2.6 Spectrophotometric Estimation of β‐Carotene‐Equivalent Carotenoid Content

We estimated β‐carotene‐equivalent carotenoid content using a validated spectrophotometric method, modified for dairy matrices (Rodriguez‐Amaya 2001). About 5 g of well‐stirred yoghurt was homogenized with 50 mL cold acetone using a high‐speed homogenizer for 2–3 min. The slurry was vacuum‐filtered with Whatman No. 1 filter paper, and the residue was rinsed several times with acetone until colorless to extract all the pigment (Zhao et al. 2024).

The acetone extracts were pooled and placed into a separating funnel with 50 mL of petroleum ether. Distilled water was added to aid phase separation (and to remove acetone); the aqueous phase was discarded, and the washing step was repeated with distilled water until the aqueous phase turned colorless. The ether layer (carotenoid extract) was collected, dried over anhydrous sodium sulfate, and brought up to a known volume. Absorbance was measured at 470 nm in a UV–vis spectrophotometer using petroleum ether as a blank, and β‐carotene concentration was calculated using the specific extinction coefficient and a standard curve prepared from a pure β‐carotene standard (Zhao et al. 2024). Calibration curves were prepared using β‐carotene standards ranging from 0.5 to 10 μg mL^−1^ (*R* ^2^ > 0.99). The limit of detection (LOD = 0.2 μg mL^−1^) and limit of quantification (LOQ = 0.5 μg mL^−1^) were determined according to standard analytical procedures.

### 2.7 Analytical Performance of the β‐Carotene‐Equivalent Carotenoid Method

Linearity, accuracy, precision, sensitivity, and recovery were used to validate the analytical method for quantification of β‐carotene (Ermer 2025). The calibration standards were prepared from standard solutions of β‐carotene in the concentration range of 0.5–10 μg/mL. The calibration curve was found to be linear with a regression coefficient (*R* ^2^) of more than 0.99. The limit of detection (LOD) and limit of quantification (LOQ) were determined by standard analytical procedures and were found to be 0.2 and 0.5 μg/mL, respectively.

The accuracy of the extraction and quantification procedure was evaluated using recovery experiments by spiking yoghurt matrix samples with known concentrations of β‐carotene standard. Three fortification levels (1, 5, and 10 μg/mL) corresponding to low, medium, and high concentration ranges were analyzed. The percentage of recovery was determined by dividing the measured concentration of β‐carotene by the added concentration of β‐carotene. The recovery was 96.4% on average.

Intra‐day and inter‐day repeatability studies were used to assess method precision. Intra‐day precision was obtained by repeating the analysis on the same day, and inter‐day precision was obtained by repeating the analysis on different analytical days. The results were reported as relative standard deviation (RSD%). The intra‐day and inter‐day RSD values were 4.26% and 4.16%, respectively, indicating good analytical repeatability and reproducibility.

### 2.8 Preparation of Extract for Antioxidant Assays

For determination of antioxidant activity (DPPH), total phenolic content (TPC), and total flavonoid content (TFC), 1 g of well‐homogenized yoghurt sample was extracted with 10 mL of ethanol (sample‐to‐solvent ratio 1:10, w/v), yielding a stock extract concentration equivalent to 100,000 ppm (100 mg/mL). The mixture was subjected to shaking extraction for 18 h at room temperature in the dark to prevent photodegradation of light‐sensitive bioactive compounds. Following extraction, the mixture was centrifuged at 6000 rpm for 10 min, and the supernatant was filtered through Whatman No. 1 filter paper (11 μm pore size) to obtain a clear extract. Working solutions of 50, 100, 200, 400, and 800 ppm were prepared by diluting appropriate aliquots (5, 10, 20, 40, and 80 μL, respectively) of the stock extract to a final volume of 10 mL with ethanol, and these were used for the DPPH radical scavenging assay.

### 2.9 Determination of Antioxidant Activity

A volume of 2 mL of ethanol DPPH solution was combined with 2 mL of extract solution at several concentrations (50, 100, 200, 400, 800 ppm). Working extract solutions of 50, 100, 200, 400, and 800 μg/mL were prepared. Equal volumes of each working extract solution (2 mL) and DPPH solution (2 mL) were mixed. Therefore, the stated 50–800 μg/mL concentrations refer to the extract solutions before mixing, corresponding to final extract concentrations of 25–400 μg/mL in the assay mixture. The resulting mixtures were vigorously stirred for 15 s. A blank solution was prepared the same way. In the blank, a control was prepared using DPPH solution and ethanol without sample extract. The solutions were then allowed to stand in the dark at room temperature for 30 min to complete the reaction. After 30 min, absorbance was measured against the blank at 517 nm (Abdullah et al. 2024). The DPPH radical scavenging percentage was calculated as [(*A*_0_ – *A*_1_)/*A*_0_] × 100, where *A*_0_ = Blank Absorbance (DPPH + Ethanol) and *A*_1_ = Sample Absorbance (DPPH + Extract) (Brand‐Williams et al. 1995; Supapvanich et al. 2025).

The DPPH radical‐scavenging activity at an 800 μg/mL working extract concentration before 1:1 mixing with DPPH solution corresponds to a final extract concentration of 400 μg/mL in the reaction mixture.

IC_50_ (based on pre‐mixing working extract concentrations) values were calculated only when 50% inhibition was observed in the concentration range tested. When 50% inhibition was not achieved at the highest concentration tested (800 ppm), the IC_50_ was not extrapolated beyond the range of tested concentrations and was reported as > 800 ppm.

### 2.10 Determination of Total Phenolic Content (TPC)

8.5 mL of distilled water, 0.5 mL of the Folin–Ciocalteu phenol reagent, and 0.5 mL of the sample were mixed. After that, the mixture was allowed to sit at room temperature for 30 min. A 1 mL solution of 35% sodium carbonate was then added. After vigorously agitating the solution and allowing it to equilibrate for 20 min at room temperature, the optical density was measured at 765 nm. TPC was calculated from the gallic acid calibration curve and expressed as mg gallic acid equivalents per 100 g of fresh yoghurt (mg GAE/100 g), taking into account the original sample mass, extraction volume, and dilution factor (Abdullah et al. 2024).

### 2.11 Determination of Total Flavonoid Content (TFC)

In a 10 mL volumetric flask with 4 mL of water, 1 mL of extract or catechin standard solution was added. 0.3 mL of 5% NaNO_2_ and 0.3 mL of 10% AlCl_3_ were added to the flask. After 6 min, 2 mL of 1 M NaOH was added, followed by H_2_O to bring the volume to 10 mL. The absorbance was measured at 510 nm using a prepared blank reagent after the solution had been mixed. The total flavonoid content was indicated as mg catechin equivalents (CE)/100 g of fw (de Souza et al. 2014). Total flavonoid content was calculated from a catechin standard calibration curve and expressed as mg catechin equivalents (CE) per 100 g of yoghurt (fresh weight).

### 2.12 Microbiological Analysis

Selected microbiological indicators were evaluated during refrigerated storage. Coliforms and *Escherichia coli* were assessed using the microbiological procedures described below, and results were expressed relative to the analytical detection limit. No total viable count, yeast and mold count, or starter‐culture viability assessment was performed; therefore, the microbiological findings were interpreted only with respect to the evaluated indicators. Yoghurt samples were serially diluted in sterile peptone water.

#### 2.12.1 *Coliforms* and *Escherichia coli* ( *E. coli* )

*Coliforms and E. coli* were counted on Violet Red Bile Agar and MacConkey Agar after incubation at 37°C for 24–48 h, in agreement with ISO 4832; suspicious colonies were tested using EC Broth.

Microbial counts were expressed as log CFU/g or mL, and results were evaluated throughout storage to determine the effect of enrichment on microbial stability (Matar et al. 2025).

### 2.13 Sensory Evaluation

Sensory evaluation was carried out as described for high‐impact drinking yoghurt‐based studies (Bolini et al. 2025; Pohjanheimo and Sandell 2009). A consumer panel comprising 30 adult participants (15 males and 15 females, aged 18–40 years) was recruited from students and staff. The tests were carried out in a sensory laboratory with illumination and temperature control. The sensory evaluation was carried out only after the preparation of the drinking yoghurt formulations. All three formulations (A, B, and C) were served at 4°C in coded identical containers in randomized and balanced order, and each panelist evaluated all three formulations. Samples were coded with three‐digit random numbers and served at refrigerated temperature under controlled conditions. Panelists rinsed their mouths with water between samples.

Panelists rated appearance/color, aroma, taste, and texture/mouthfeel using a 9‐point hedonic scale (1 = dislike extremely, 9 = like extremely), as described by Molina et al. and others for carotenoid‐ or plant‐enriched yogurts. Water was provided for mouth rinsing between samples (Inocente‐Camones et al. 2022).

The sensory evaluation protocol received ethical clearance from the Institutional Review Board (IRB), Faculty of Health and Life Sciences, Daffodil International University, Bangladesh (Ref. No. FHLS‐REC/DIU/2025/0011, dated June 12, 2025).

### 2.14 Physicochemical Parameters (pH)

The pH was determined three times for each formulation at each storage time point. These measurements were technical replicates and were used to evaluate analytical repeatability. The pH was measured at 25°C using a bench‐top pH meter (daily calibrated with buffer solutions at pH 4.0 and 7.0).

### 2.15 Statistical Analysis

The three drinking yoghurt formulations were produced in a single production run. Triplicate measurements were technical replicates for proximate composition, β‐carotene‐equivalent carotenoid content, DPPH radical‐scavenging activity, TPC, TFC, and pH. These data were therefore summarized descriptively as mean ± standard deviation (SD), and no inferential comparison was made among formulations based on these technical replicates. The sensory evaluation was conducted with all 30 participants in the same session; thus, a within‐subject design was used. An appropriate repeated‐measures procedure was used to analyze sensory scores for each attribute. The level of statistical significance was defined as *p* < 0.05. IBM Statistical Package for the Social Sciences (SPSS) version 25 was utilized to conduct descriptive analysis (mean ± standard deviation) for proximate and bioactive composition, and one‐way repeated‐measures ANOVA was used to analyze sensory results. Besides, the statistical packages ggplot2 and tidyr were used to visualize pH values using RStudio (version 4.3.2).

## 3 Results

Proximate composition of fortified drinking yoghurt varied for the different formulations. Moisture content ranged from 81.72% ± 0.07% (Sample C) to 84.27% ± 0.06% (Sample B). The protein content of sample C (3.75% ± 0.09%) and carbohydrate (10.33% ± 0.30%) were the highest, while the protein, fat, and carbohydrate content were the lowest in sample B (0.63% ± 0.05%). Sample C, on average, had a higher nutrient‐dense profile with slightly more protein, fat, and carbohydrate as compared to Sample B, which had the highest moisture and ash profile (Table 1).

**TABLE 1:** Proximate composition of fortified drinking yoghurt.

Proximate component	Sample A (%)	Sample B (%)	Sample C (%)
Moisture	82.68 ± 0.12	84.27 ± 0.06	81.72 ± 0.07
Ash	0.53 ± 0.04	0.63 ± 0.05	0.53 ± 0.01
Protein	3.56 ± 0.05	3.07 ± 0.07	3.75 ± 0.09
Fat	3.47 ± 0.06	3.15 ± 0.05	3.67 ± 0.16
Carbohydrate	9.76 ± 0.09	8.88 ± 0.21	10.33 ± 0.30

*Note:* Here, values are the mean ± SD of three technical measurements from a single production run.

Table 2 depicts that the three drinking yoghurt formulations were different in sensory scores for appearance, aroma, taste, and texture (*p* < 0.001). Sample A showed the highest mean scores for appearance (7.63 ± 1.16), aroma (8.00 ± 1.05), taste (8.07 ± 0.98), and texture (7.90 ± 1.06), whereas Sample C showed the lowest mean scores for all four attributes. The results show that formulation A had the most acceptable sensory rating of the evaluated formulations.

**TABLE 2:** Sensory evaluation scores of the fortified drinking yoghurt formulations.

Sensory attribute	Sample A	Sample B	Sample C	*p*‐value
Appearance	7.63 ± 1.16	7.20 ± 1.16	6.57 ± 1.36	< 0.001
Aroma	8.00 ± 1.05	7.30 ± 1.39	6.63 ± 1.30	< 0.001
Taste	8.07 ± 0.98	6.80 ± 1.10	6.10 ± 1.00	< 0.001
Texture	7.90 ± 1.06	6.93 ± 1.08	6.60 ± 1.10	< 0.001

*Note:* Values are presented as mean ± SD (*n* = 30) based on a 9‐point hedonic scale (1 = dislike extremely; 9 = like extremely).

Figure 1 shows that the pH of all samples decreased gradually over the 35‐day storage period, indicating that the acidification process continued. During storage, sample B maintained a relatively high pH, while sample C maintained a relatively low pH. Overall, the pH values were 4.5 (sample B at the initial and Day 7) and 4.0 (sample C on Day 35).

**FIGURE 1:**
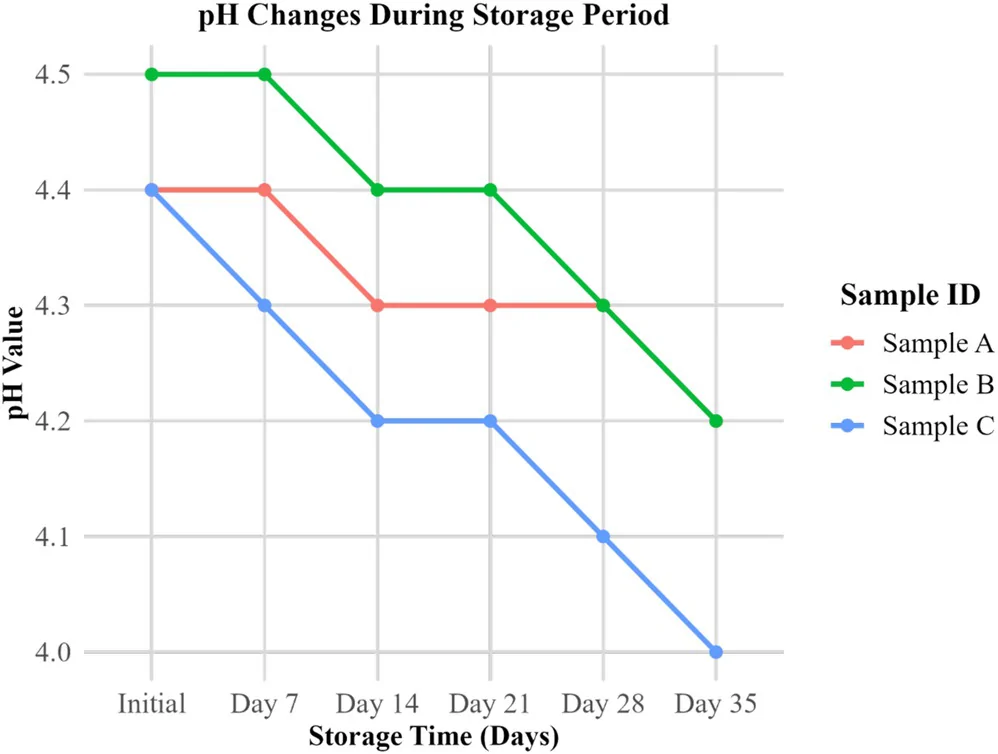
pH value of fortified drinking yoghurt.

Table 3 shows the bioactive composition of fortified beverage samples A, B, and C. Differences in the observed mean values were noted among the formulations for DPPH radical‐scavenging activity, total phenolic content, and total flavonoid content. At the same time, β‐carotene equivalent carotenoid content ranged from 4.5 to 4.9 mg/100 g among the formulations.

**TABLE 3:** Bioactive composition of fortified drinking yoghurt.

Bioactive compounds	Sample A	Sample B	Sample C
β‐carotene‐equivalent carotenoid content (mg/100 g)	4.7 ± 0.2	4.5 ± 0.3	4.9 ± 0.2
DPPH radical‐scavenging activity (% inhibition)	51.2 ± 1.7	56.1 ± 2.1	47.4 ± 2.2
IC_50_ (ppm)	668.9	429.4	> 800
TPC (mg GAE/100 g)	50.2 ± 1.6	51.4 ± 1.5	38.35 ± 1.15
TFC (mg CE/100 g)	11 ± 0.9	14 ± 1	9.2 ± 0.3

*Note:* Values are the mean ± SD of three technical measurements from a single production run, except for IC_50_ values. Technical replicates were used to assess analytical repeatability and were not treated as independent experimental units. IC_50_ of Sample C was approximately 848.67 ppm based on extrapolation; however, since 50% inhibition was not achieved within the tested concentration range, the IC_50_ was conservatively reported as > 800 ppm.

The highest antioxidant activity was observed at 800 ppm extract concentration in sample B (56.1%) and the lowest in sample C (47.4%). Values represent percentage inhibition at 800 ppm extract concentration. Based on the concentration‐response data, the estimated IC_50_ values for Samples A and B were 668.9 and 429.4 ppm, respectively. Sample C exhibited 47.4% ± 2.2% DPPH radical‐scavenging activity at the highest concentration tested (800 ppm) and thus failed to achieve 50% inhibition within the tested concentration range. Therefore, its IC_50_ was not extrapolated beyond the range of experimental concentrations, but was reported as > 800 ppm. Similarly, TPC ranged from 38.35 to 51.4 mg GAE/100 g, with sample B the highest (51.4 mg GAE/100 g) and sample C the lowest (38.35 mg GAE/100 g). Overall, sample B exhibited high antioxidant capacity and high phenolic and flavonoid content, whereas sample C exhibited relatively low bioactive compounds.

Table 4 represents the analytical performance of the spectrophotometric method for β‐carotene‐equivalent carotenoid estimation. The validation results showed that the spectrophotometric method used was reliable for the estimation of β‐carotene‐equivalent carotenoids in fortified drinking yoghurt. The calibration curve showed excellent linearity over the concentration range of 0.5–10 μg/mL (*R* ^2^ > 0.99). The sensitivity of the method was confirmed by the low LOD (0.2 μg/mL) and LOQ (0.5 μg/mL). The recovery analysis showed 96.4% recovery, which was satisfactory for the complex dairy matrix. In addition, the RSD values obtained for intra‐day (4.26%) and inter‐day (4.16%) precision were low, indicating good analytical repeatability and reproducibility.

**TABLE 4:** Validation performance of β‐carotene quantification.

Parameter	Result
Analytical wavelength	470 nm
Extraction method	Acetone extraction followed by petroleum ether partitioning
Calibration range	0.5–10 μg/mL
Regression coefficient (*R* ^2^)	> 0.99
LOD	0.2 μg/mL
LOQ	0.5 μg/mL
Recovery	96.4%
Spiking levels	1, 5, and 10 μg/mL
Intra‐day precision (RSD%)	4.26%
Inter‐day precision (RSD%)	4.16%

Table 5 denotes that no *coliform* bacteria or *E. coli* were detected in the yoghurt at any time point up to 35 days of storage (all samples were negative). This indicates microbiologically acceptable under the refrigerated storage conditions tested. After 35 days of storage at 4°C, both coliforms and *E. coli* were absent in the improved drinking yoghurt samples, indicating the absence of detectable coliforms and *E. coli* under the tested conditions within the evaluated parameters. The highest and lowest observed values for both microorganisms were less than 1 CFU/mL, indicating no detectable growth at any stage of storage.

**TABLE 5:** Presence of *Coliform* and *E. coli* in the developed drinking yoghurt samples during refrigerated storage.

The time period of storage (at 4°C)	Sample A		Sample B		Sample C	
	*Coliform*	*E. coli*	*Coliform*	*E. coli*	*Coliform*	*E. coli*
Initial	< 1.0 (ND)	< 1.0 (ND)	< 1.0 (ND)	< 1.0 (ND)	< 1.0 (ND)	< 1.0 (ND)
Day 07	< 1.0 (ND)	< 1.0 (ND)	< 1.0 (ND)	< 1.0 (ND)	< 1.0 (ND)	< 1.0 (ND)
Day 14	< 1.0 (ND)	< 1.0 (ND)	< 1.0 (ND)	< 1.0 (ND)	< 1.0 (ND)	< 1.0 (ND)
Day 21	< 1.0 (ND)	< 1.0 (ND)	< 1.0 (ND)	< 1.0 (ND)	< 1.0 (ND)	< 1.0 (ND)
Day 28	< 1.0 (ND)	< 1.0 (ND)	< 1.0 (ND)	< 1.0 (ND)	< 1.0 (ND)	< 1.0 (ND)
Day 35	< 1.0 (ND)	< 1.0 (ND)	< 1.0 (ND)	< 1.0 (ND)	< 1.0 (ND)	< 1.0 (ND)

*Note:* Here, ND = not detected; detection limit < 1 CFU/mL.

## 4 Discussion

The present study proved that the addition of yoghurt, beetroot pulp, and orange juice to drinking yoghurt was successful in improving its physicochemical properties and increasing its useful value, without compromising microbiological safety. Proximate composition, sensory attributes, pH changes during storage, bioactive‐related measurements, and microbiological indicators showed variations among the three formulations, with the observed differences depending on the measured parameters. Behind the changes, dairy proteins (mainly caseins and whey proteins) could perform a background mechanism, as they can bind plant polyphenols mainly by non‐covalent interactions, involving mainly hydrophobic and electrostatic interactions, and the binding depends on the structure of both forms, as well as pH and temperature (Yildirim‐Elikoglu 2019). These milk proteins are regarded as natural carriers for enhancing the bioavailability and bio accessibility of polyphenols (Mao et al. 2024; Tosif et al. 2021; van de Langerijt et al. 2023).

The variations in proximate composition between the formulations may have resulted from the compositional properties and subsequent modifications to the yoghurt matrix. Sample C (255 g yoghurt +15 g beetroot pulp +30 mL orange juice) contained comparatively high levels of protein, fat, and carbohydrates, while Sample B (225 g yoghurt +45 g beetroot pulp +30 mL orange juice) had the least. This difference likely happened because the amount of yoghurt and beetroot pulp varied between the samples. The observed increase in almost all proximate composition (protein, fat, and ash) contents with increasing yoghurt proportion can be attributed to the higher proportion of milk solids, as yoghurt is a major source of milk proteins, milk fat, and minerals (Walstra et al. 2005; Muehlhoff et al. 2013; Tamime and Robinson 2007). Because the addition of beetroot in fortified yoghurt may decline fat and protein levels, e.g., a small increase in beetroot juice can drop protein percentages by roughly 0.2%–0.3% (Arvelli 2025). Another study showed that the inclusion of beetroot powder and yoghurt significantly increased protein levels, whereas fat levels showed no significant difference (Nyamete and Mongi 2017). Besides, the addition of dietary solids, naturally occurring carbohydrates, minerals, and structural elements, along with the incorporation of beetroot pulp, improved the moisture‐to‐total solids ratio. Changes in moisture distribution are particularly important in fermented dairy products because water‐binding interactions among milk proteins, plant fibers, and polysaccharides affect product consistency and stability (Li et al. 2023; Zhang et al. 2024). The findings are in line with earlier research indicating the enhanced nutritional quality of dairy products enriched with plant‐based foods (Sukhikh et al. 2019; Thakur et al. 2022).

Consumer acceptance is a critical determinant for the commercial success of functional dairy products. The sensory results indicate that a moderate incorporation of beetroot pulp and orange juice resulted in the most desirable balance between functional enhancement and palatability (Sample A). Sample C received lower taste scores which may be associated with stronger beetroot‐derived flavor intensity and altered mouthfeel., mainly due to geosmin and related volatile compounds. Sample C received the lowest taste score among the evaluated formulations. However, because Sample C contained the lowest proportion of beetroot pulp and specific flavor descriptors such as earthiness were not directly assessed, the reason for its lower taste score cannot be established from the present data. The observed sensory differences may reflect the overall formulation balance and interactions among yoghurt, beetroot pulp and orange juice; however, specific flavor attributes were not evaluated in this study (Lu et al. 2003). Compounds characteristic of beetroot, which contribute to its identity, may reduce consumer acceptance in excessive concentrations. The lower preference associated with formulations with higher levels of beetroot pulp is therefore likely related to a stronger perception of earthy flavor, a higher color intensity, and possible changes in the mouthfeel (Dikme 2025). Comparable findings have been reported in previous studies, where higher concentrations of beetroot or vegetable powders led to undesirable earthy flavor, darker color, and textural changes that negatively impacted consumer acceptance (Alam et al. 2026). The results obtained so far emphasize the importance of formulation to achieve a balance between consumer preferences and nutritional benefits. Furthermore, the beneficial sensory performance of formulations containing both orange juice and beetroot may be related to the flavor masking properties of chemicals derived from citrus. Natural sugars, acids, and volatile aromatics from orange juice can increase flavor complexity and reduce the perception of undesirable earthy notes (Dikme 2025).

During refrigerated storage, the pH trajectory obtained is in line with the common post‐acidification curve of fermented dairy products. Lactic acid bacteria retain residual metabolic activity during chilled storage, which continues to generate acid and gradually reduces yoghurt pH (Guan et al. 2025). This leads to a gradual formation of organic acids and a gradual fall in pH. The addition of orange juice and beetroot pulp did not appear to affect fermentation stability, as the pH remained within the typical range for drinking yoghurt (Colakoglu and Gursoy 2011). The present study's findings are consistent with previous research, which has shown that the use of beetroot pulp does not affect the stability of drinking yoghurt (Adjei et al. 2024; Elizabeth and Onyinyechi 2012). Our results differ from those reported by Nasim and Khalid; in their study, the beetroot extract emulsion treatments significantly influenced pH and acidity (Nasim and Khalid 2026).

In comparison with sample A (240 g yoghurt + 30 g beetroot pulp) and C (255 g yoghurt + 15 g beetroot pulp), Sample B (225 g yoghurt + 45 g beetroot pulp) showed a considerable improvement in antioxidant activity, total phenolic content, and total flavonoid content, implying that the sample has a greater amount of free radical scavenging ability because of the increased percentage of beetroot‐derived bioactive compounds (Soutelino et al. 2023). Prior studies revealed that even low concentrations (1%–3%) of beetroot juice lead to a statistically significant (*p* < 0.05) rise in free radical scavenging (DPPH%) and antioxidant activity (Salman et al. 2024).

A widely used method for the estimation of carotenoids is spectrophotometric measurement at 470 nm, but this method should be used with caution in complex food products. The developed yoghurt formulation included beetroot pulp and orange juice, which could provide further pigments such as the betalains and other carotenoids found in citrus fruits, which may have overlapping absorbance properties.

The present method used acetone extraction and petroleum ether partitioning to reduce potential interference. This solvent separation step is useful for the extraction of lipid‐soluble carotenoids and minimizes the contribution of more polar pigments. Thus, the absorbance measured is not the concentration of β‐carotene but the β‐carotene equivalent carotenoid content.

The extraction and measurement procedure was reliable in the studied dairy matrix, as the acceptable recovery (96.4%) and precision (< 5% RSD) were obtained. However, further research is suggested to identify individual carotenoids and to profile all the carotenoids by chromatographic methods like HPLC‐DAD or LC–MS.

The microbiological analysis revealed that no coliforms or *E. coli* were detected in any sample during the 35 days of storage at 4°C. The absence of these pathogenic indicators indicates good hygienic processing, fermentation control, and refrigerated storage preservation. The test results are in good alignment with the accepted safety standards. For instance, the findings are in line with the International Organization for Food Microbiology (IOFM) and regulatory agencies such as the FDA and EFSA, which have set limits for coliforms and *E. coli* in refrigerated food products at < 10 CFU/g (Abdullahi and Abdulkareem 2010). The findings of this study indicate that these pathogens are completely absent, indicating satisfactory results for the evaluated microbial indicators. For instance, a study (Damunupola et al. 2014) found similar microbiological stability in fortified yoghurt when stored under similar refrigerated conditions, where no coliforms or *E. coli* were detected after 21 days. Likewise, Jovanović et al. (2021) showed that the growth of pathogenic bacteria in yoghurt can be effectively inhibited by refrigerating at 4°C. Other studies, however, have reported different results, with coliforms being detected in beetroot‐fortified yoghurt after just 2 days of storage (Elizabeth and Onyinyechi 2012), indicating that the hygienic conditions of the raw materials or the fermentation process may influence the microbiological results.

Overall, this study demonstrates that the fortification of drinking yoghurt with beetroot pulp and orange juice is a promising approach for the development of functional dairy beverages with enhanced bioactive properties, acceptable sensory quality, and absence of detectable coliforms and *E. coli* under the evaluated conditions. The most palatable formulation was Sample A in terms of sensory quality, and Sample B had better antioxidant potential, indicating that careful optimization of ingredient proportions is crucial to maximize health‐promoting attributes whilst maintaining consumer acceptability and selected quality characteristics.

But there are a few caveats to note. The microbiological evaluation was limited to coliform detection, and total viable counts (TVC) were not quantified, so the microbial load and dynamics of lactic acid bacteria during storage remain incompletely characterized. In addition, the nutritional analysis was limited to macronutrients (fat, protein, and carbohydrates). It did not include micronutrient profiling (such as vitamins, minerals, and specific phytochemicals), which reduces the statistical robustness and depth of the nutritional claims. Comprehensive TVC monitoring and extended microbiological profiling should be included in future investigations to better understand microbial stability over time. Furthermore, the nutritional analysis was conducted only to the extent of proximate composition and did not include a more in‐depth micronutrient profile, including vitamins and minerals. This restriction limits the nutritional characterization. Future studies should include independently replicated production batches, a thorough microbiological investigation, detailed micronutrient and phytochemical analysis, and a wider consumer analysis. They would give a more comprehensive characterization of the functional benefits of beetroot‐orange fortified drinking yoghurt.

## 5 Conclusion

This study characterized three drinking yoghurt formulations containing orange juice and different proportions of beetroot pulp. Therefore, the present findings should be interpreted as preliminary formulation‐level observations. Among the formulations evaluated, Sample A showed the highest observed mean sensory scores, whereas Sample B showed the highest observed DPPH radical‐scavenging activity, total phenolic content, and total flavonoid content. β‐Carotene‐equivalent carotenoid content showed a relatively narrow range among the formulations, and pH gradually decreased during refrigerated storage. Coliforms and *E. coli* were not detected above the method detection limit during the 35‐day storage period. However, the study was based on a single production run, lacked an unfortified control, and evaluated only selected microbiological indicators. Therefore, independently replicated production trials, broader microbiological and physicochemical shelf‐life analyses, compound‐specific carotenoid profiling, and larger consumer studies are required before conclusions regarding product stability, health benefits, scale‐up, or commercialization can be made.

## Author Contributions

**Md Masudul Islam Khan:** conceptualization, methodology, data curation, investigation, resources, writing – original draft, writing – review and editing. **Md Noitik Ali:** conceptualization, methodology, data curation, investigation, project administration, writing – original draft, writing – review and editing, resources. **Ayshee Tabassum:** writing – original draft, writing – review and editing. **Devpriya Bhattacharjee:** writing – original draft, writing – review and editing. **Shahin Alam:** writing – original draft, writing – review and editing. **Md Shahedul Islam:** formal analysis, supervision, visualization, software, writing – review and editing.

## Funding

The authors have nothing to report.

## Disclosure

All authors have read and approved the final version of the manuscript. Md Shahedul Islam, corresponding author and manuscript guarantor, had full access to all data in this study and takes full responsibility for the integrity and accuracy of the data analysis.

## Ethics Statement

The sensory evaluation protocol received ethical clearance from the Institutional Review Board (IRB), Faculty of Health and Life Sciences (FHLS), Daffodil International University (DIU), Bangladesh, under Ref. No. FHLS‐REC/DIU/2025/0011, dated 12 June 2025.

## Consent

Written informed consent was obtained from all panelists before sensory evaluation. Panelists were volunteers; panelists could withdraw at any time, and no identifiable personal, clinical, or sensitive information was collected.

## Conflicts of Interest

The authors declare no conflicts of interest.

## Data Availability

The data that support the findings of this study are available from the corresponding author upon reasonable request.
